# A review of methodology for sample size calculations in cluster randomised trials

**DOI:** 10.1186/1745-6215-12-S1-A23

**Published:** 2011-12-13

**Authors:** Clare Rutterford, Sandra Eldridge, Andrew Copas

**Affiliations:** 1 Centre for Primary Care and Public Health, Queen Mary University of London, London, E1 2AB, UK; 2 Hub for Trials Methodology Research, MRC Clinical Trials Unit, London, UK

## Objectives

To produce a thorough review of the existing state of knowledge on sample size calculations for cluster randomised trials (CRT’s) and to identify gaps in the knowledge.

## Methods

A systematic review is being conducted of sample size methodology for cluster randomised trials. The sources for the search include electronic databases PubMed and Web of Science, key text books on cluster randomised trials and discussions with experts in the field.

The search strategy involves a compliment of Medical Subject Headings and free text terms to aid a comprehensive search. The references of papers eligible for the review will also be searched and a search on the first author conducted. This process will continue until no more additional papers are located.

This work forms the beginning of a PhD research project.

## Results

Of 8697 citations obtained from PubMed and Web of Science, the majority have currently been assessed for eligibility into the review and 57 papers so far identified for inclusion.

The majority of papers discuss sample size for continuous or binary outcomes, with four papers discussing time to event outcomes. In terms of the analysis method used, most assume a random effects analysis (cluster specific approach) or a cluster level analysis, with fewer papers assuming a generalized estimating Equation (population averaged approach) methodology.

An emerging theme, discussed in six papers, is sample size methodology for 3-level cluster randomised trials, where we may randomise clinics (level 3) and each clinic will treat multiple subjects (level 2 units) who in turn are measured on repeated occasions (level 1 units).

Eight papers consider sample size calculations for trials with varying cluster sizes. These papers account for the loss in power due to varying cluster sizes through an examination of the relative efficiency of unequal versus equal cluster sizes or by proposing an appropriate design effect to account for this loss for both continuous and binary outcomes.

Sample size for alternative trial designs such as cross-over trials, stepped wedge designs, testing for non-inferiority, stratified, and matched designs were identified. Papers covering adjustments to sample size for dealing with non-compliance or attrition, accounting for the use of cluster or person level covariates and dealing with imprecision in the estimate of the intracluster correlation coefficient (ICC) were identified.

## Conclusion

We will provide the results of the search and preliminary insight into potential gaps in the knowledge.

